# Terahertz time-domain spectroscopy for powder compact porosity and pore shape measurements: An error analysis of the anisotropic bruggeman model

**DOI:** 10.1016/j.ijpx.2021.100079

**Published:** 2021-04-27

**Authors:** Moritz Anuschek, Prince Bawuah, J. Axel Zeitler

**Affiliations:** a Department of Chemical Engineering and Biotechnology, University of Cambridge, Philippa Fawcett Drive, CB3 0AS Cambridge, UK; b Department of Pharmacy, Pharmaceutical Technology and Biopharmaceutics, Ludwig-Maximilians-University, Butenandtstraße, Munich, Germany

**Keywords:** Pharmaceutical tablet, Terahertz, Pore structure, Density distribution, Anisotropy, Bruggeman model, *a*_1_, Gradient of the depolarisation factor as a function of porosity, *a*_2_, Y-intercept of the depolarisation factor as a function of porosity, AB-EMA, Anisotropic Bruggemen model, API, Active pharmaceutical ingredient, *c*, Speed of light, *D*, Tablet diameter, *f*, Porosity, *f*_1_, Lowest porosity in a set of tablets, *H*, Tablet thickness, Ibu, Ibuprofen formulation, *L*, Depolarisation factor, *L*_1_, Depolarisation factor at the lowest porosity, *L*_fit_, Estimation of the depolarisation factor based on a fitting model, *L*_l/u_, Lower/upper bound of the depolarisation factor, *L*_max/min_, Maximal/minimal depolarisation factor in the simulation of a tablet set, Lac, Lactose, *M*, Tablet mass, MCC, Microcrystalline cellulose, *n*, Refractive index, $\tilde{n}$, Complex refractive index, *n*_eff_, Effective refractive index, *n*_eff, 1_, Effective refractive index at the lowest porosity, *n*_eff, l/u_, Lower/upper Wiener bound for *n*_eff_, *n*_s_, Intrinsic refractive index of the solid fraction, *n*_s, c_, *n*_s_ estimated with accounting for absorption, *n*_s, fit_, Estimation of the intrinsic refractive index based on a fitting model, *n*_eff, l/u_, Lower/upper margin for *n*_s_, *p*, Polar axis of a spheroid, parallel to the wavevector, *q*, *r*, Equatorial axes of a spheroid, perpendicular to the wavevector, RMSE, Root-mean squared error, tablename Str, Starch, THz-TDS, Terahertz time-domain spectroscopy, *α*_eff_, Effective absorption coefficient, ${\tilde{\epsilon }}_{\mathrm{eff}}$, Effective complex dielectric permittivity, ${\tilde{\epsilon }}_{s}$, Complex dielectric permittivity of the solid fract, *κ*, Extinction coefficient, *κ*_eff_, Effective extinction coefficient

## Abstract

Terahertz time-domain spectroscopy (THz-TDS) is a novel technique which has been applied for pore structure analysis and porosity measurements. For this, mainly the anisotropic Bruggeman (AB-EMA) model is applied to correlate the effective refractive index (*n*_eff_) of a tablet and the porosity as well as to evaluate the pore shape based on the depolarisation factor *L*. This paper investigates possible error sources of the AB-EMA for THz-TDS based tablet analysis. The effect of absorption and tablet anisotropy – changes of pore shape with porosity and density distribution – have been investigated. The results suggest that high tablet absorption has a negligible effect on the accuracy of the AB-EMA. In regards of tablet anisotropy the accuracy of the porosity determination is not impaired significantly. However, density distribution and variations in the pore shape with porosity resulted in an unreliable extraction of the tablet pore shape. As an extension of the AB-EMA a new concept was introduced to convert the model into bounds for *L*. This new approach was found useful to investigate tablet pore shape but also the applicability of the AB-EMA for an unknown set of data.

## Graphical abstract

**Figure:**
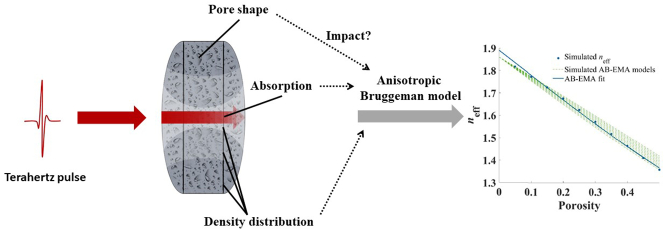

## 1 Introduction

Tablets are considered the most convenient way to deliver active pharmaceutical ingredients (APIs) to a patient given their ability to achieve accurate dosing, long shelf life and cost-effective production (Desai et al., 2016). Porosity and pore anisotropy are recognised as important parameters that influence tablet disintegration and API dissolution (Markl et al., 2018a). Important precursors of disintegration such as swelling and liquid imbibition have been found to be dependent on total tablet porosity (Yassin et al., 2015a, Yassin et al., 2015b). However, it is also well known that total porosity alone is not sufficient to fully describe the liquid imbibition process (Berg, 2014) and that pore anisotropy including tortuosity and connectivity need to be considered as well amongst other factors (Markl et al., 2018b).

Terahertz time-domain spectroscopy (THz-TDS) has been suggested as a fast and non-destructive technique for porosity and pore shape measurements (Bawuah et al., 2016b; Bawuah and Peiponen, 2016; Bawuah et al., 2016c). The use of non-ionising radiation and the advantage of fast (sub-second) measurement acquisition makes it ideal for in-line or at-line control applications (Bawuah et al., 2020). Furthermore, most pharmaceutical excipients are at least semi-transparent at terahertz frequencies allowing for measurements in transmission mode (Zeitler et al., 2007). Due to the nature of the measurements being performed in the time-domain, the refractive index can be extracted from the measurements without relying on more complicated models such as the Kramers-Kronig relations, which may require further interpretation or constraints applied to succeed (Bawuah et al., 2014).

The refractive index at terahertz frequencies has been found to correlate with the porosity (Bawuah et al., 2014, Bawuah et al., 2016b; Juuti et al., 2009). In addition to being able to measure total porosity, by using different physical models, it is possible to gain further insight into the pore structure of tablets. Such advanced analyses of the terahertz data is implemented using models from effective medium theory. The anisotropic Bruggeman model (AB-EMA) is the most commonly used approach and this method has been demonstrated to allow for the extraction of pore shape information in addition to the tablet porosity (Bawuah et al., 2020).

The focus of this work will be to provide an error analysis of the AB-EMA model for THz-TDS based porosity and pore shape analysis. It will critically examine the impact and sensitivity of the following assumptions that are required when applying the model to experimental data on the accuracy of the results:

I) The AB-EMA utilises the complex dielectric permittivity ($\tilde{\epsilon }$) which is defined by $\tilde{\epsilon }={\tilde{n}}^{2}={\left(n-\mathrm{i\kappa}\right)}^{2}$, where $\tilde{n}$ is the complex refractive index which is composed of the refractive index, *n*, and *κ*, the extinction coefficient. Thus far, all studies published in the pharmaceutical literature for using AB-EMA for pore analysis only consider *n* and the effect due to absorption from the tablet matrix is approximated to be negligible.
II) The AB-EMA method considers pores of different porosities to have the same shape, which can be described by the so-called depolarisation factor, *L*. However, the tablet compaction process is inherently anisotropic given the tool and process geometry. It is also well-known that increasing compaction pressure (i.e. decreasing porosity) can result in higher tablet anisotropy (Porion et al., 2010). The elastic and plastic deformation processes themselves are strongly dependent on the magnitude and direction of applied pressure (Lubarda, 2016). Since pores in tablets are formed by the void space between particles, deformation of one is considered to affect the form of the other.
III) In the most common implementation, the THz-TDS transmission measurement is performed in axial direction through the central volume of the tablet centre. However, in the subsequent analysis the measurement is considered to represent a function of nominal porosity for the entire volume of the tablet. This is only strictly valid in the absence of density distribution in the radial direction. Most tablets in the pharmaceutical industry are of biconvex geometry (Kadiri and Michrafy, 2013) and it is well-established that the punch geometry influences the pressure distribution in the tablet during compaction as well as during ejection (Kadiri and Michrafy, 2013; Mazel et al., 2015). Other than for flat-faced tablets this can result in density distribution for a range of common tablet shapes, such as in biconvex tablets (Sinka et al., 2004). The biconvex shape of the punch results in a pressure gradient in the radial direction from the centre to the edges. This may result in a lower pressure in the tablet centre compared to the edges during compaction and it has been shown that the punch curvature can lead to a density distribution (i.e. porosity distribution) in the radial direction of a biconvex tablet (Sinka et al., 2004; May et al., 2013).

THz-TDS has been suggested as a method suitable for application as a process analytical technology to measure and control tablet porosity (Bawuah and Peiponen, 2016; Bawuah et al., 2016c,b). In this study, we aim to provide a thorough analysis of the merits and limitations of this technique together with a discussion of the potential sources and magnitude of error and how they can be mitigated and controlled. In addition, a new concept based on the AB-EMA and the Wiener bounds effective medium approximation is presented to investigate changes in the pore shape with porosity as well as to evaluate the applicability of the AB-EMA on a set of test data.

## 2 Theory

### 2.1 The Anisotropic Bruggeman Effective Medium Approximation (AB-EMA)

The traditional Bruggeman model is a commonly used approach for EMA that was developed in the early 20th century by Bruggeman (1935). It describes the dielectric properties of a composite material (the so-called effective medium) by combining the individual dielectric properties, $\tilde{\epsilon }$, of the volume fractions occupied by the isolated materials where the minor fraction, the inclusions, are assumed to be of spherical shape and the major fraction forms a continuous phase (Bruggeman, 1935; Bawuah et al., 2020). This formalism was later developed further by including a depolarisation factor into what is now known as the AB-EMA, where inclusions are spheroids with their polar axis aligned parallel to the propagation direction of the electromagnetic field (Jones and Friedman, 2000). Eq. 1 describes the AB-EMA model for a porous two component system composed of air and a solid material, denoted by the subscript *s*, where ${\tilde{\epsilon }}_{s}$ and ${\tilde{\varepsilon }}_{\mathrm{eff}}$ denote the complex dielectric permittivities of the solid fraction and the effective medium respectively and *f* is the porosity. Here it was assumed that the complex dielectric permittivity of air is equal to 1.

(1) $$f\frac{1-{\tilde{\varepsilon }}_{\mathrm{eff}}}{{\tilde{\varepsilon }}_{\mathrm{eff}}+L\left(1-{\tilde{\varepsilon }}_{\mathrm{eff}}\right)}+\left(1-f\right)\frac{{\tilde{\varepsilon }}_{s}-{\tilde{\varepsilon }}_{\mathrm{eff}}}{{\tilde{\varepsilon }}_{\mathrm{eff}}+L\left({\tilde{\varepsilon }}_{s}-{\tilde{\varepsilon }}_{\mathrm{eff}}\right)}=0$$

The depolarisation factor, *L*, describes the overall shape of the inclusions, which in case of porous pharmaceutical tablets are the tablet pores. It can be converted into the aspect ratio of the semi-axes of the spheroid using Eq. 2 where the polar axis, *p*, is parallel and the equatorial axes, *q* and *r*, are perpendicular to the wavevector of an electromagnetic wave, with *q* = *r* (Jones and Friedman, 2000). For *p* = *q*, *L* becomes 1/3 and the inclusion takes a spherical form (and Eq. 1 results in the traditional Bruggeman model). Applying Eq. 2 results in a one dimensional, needle shaped prolate for *L* = 1 and a two dimensional, flat oblate for *L* = 0.

(2) $$L=\frac{1}{1+1.6\left(p:q\right)+0.4{\left(p:q\right)}^{2}}$$

For multiple solid components it was demonstrated that the dielectric properties of the different solid components can be combined into a single solid fraction with the average dielectric properties (Bawuah et al., 2020).

Most pharmaceutical materials exhibit relatively low absorption losses at terahertz frequencies. Therefore, it is often assumed that the complex part of the dielectric permittivity of the solid fraction can simply be neglected, resulting in ${\tilde{\epsilon }}_{s}=n_{s}^{2}$ and ${\tilde{\epsilon }}_{\mathrm{eff}}={n_{\mathrm{eff}}}^{2}$, where *n*_s_ and *n*_eff_ are the intrinsic refractive index of the combined solid fractions and the effective refractive index, respectively (Bawuah et al., 2020). Under these assumptions, Eq. 1 simplifies to Eq. 3 which is the common expression used in terahertz porosity analysis.

(3) $$f\frac{1-{n_{\mathrm{eff}}}^{2}}{{n_{\mathrm{eff}}}^{2}+L\left(1-{n_{\mathrm{eff}}}^{2}\right)}+\left(1-f\right)\frac{{n_{s}}^{2}-{n_{\mathrm{eff}}}^{2}}{{n_{\mathrm{eff}}}^{2}+L\left({n_{s}}^{2}-{n_{\mathrm{eff}}}^{2}\right)}=0$$

### 2.2 Bounds for *L*

The Wiener bounds are a common EMA developed by Wiener (1912). They describe the two extreme cases in which a composite material is formed either through serial or parallel alignment of the individual components, which are assumed to behave like planar plate capacitors. This results in an upper and lower bound for ${\tilde{\epsilon }}_{\mathrm{eff}}$ of the composite material, which again can be approximated in terms of *n*_eff_ for weakly absorbing materials (Wiener, 1912; Markl et al., 2018a).

Eqns. 4 and 5 show the upper and lower Wiener bound for the general case of a multicomponent material with *J* solid components with the corresponding mass fractions *x*_*j*_ and refractive indices *n*_*j*_.

(4) $$n_{\mathrm{eff},l}^{2}=\frac{1}{f+\sum_{j=1}^{J}\frac{x_{j}}{n_{j}^{2}}}$$

(5) $$n_{\mathrm{eff},u}^{2}=f+\sum_{j=1}^{J}x_{j}n_{j}^{2}$$

In case of a two component system of air and one solid fraction, s, Eqns. 4 and 5 simplify to Eqns. 6 and 7.

(6) $$n_{\mathrm{eff},l}^{2}=\frac{1}{f+\frac{1-f}{n_{s}^{2}}}$$

(7) $$n_{\mathrm{eff},u}^{2}=f+\left(1-f\right)n_{s}^{2}$$

Note that in this concept multiple solid fractions are again represented by one refractive index, *n*_s_, the same approach used in the previous section. For this case the Wiener bounds could in principle also be extended to their multicomponent equivalents (Eqns. 4 and 5). However, these do not conform with the AB-EMA model which only considers two phase compound materials and are therefore not further discussed in this article. We refer to an article by (Bawuah et al., 2016a) where these equations where utilised in the pharmaceutical setting using terahertz analysis.

**Fig. 1:**
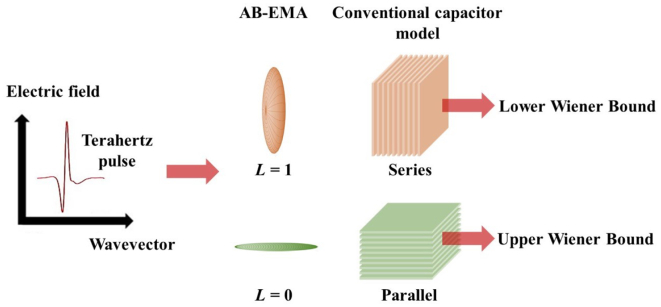
Illustration of the Wiener bounds following the conventional capacitor model and the AB-EMA inclusion shape. The spheroid represents the orientation of the air pore in the dielectric medium of the solid phase relative to the propagation direction of the electromagnetic field of the transmitted terahertz pulse.

Fig. 1 illustrates the concept of the upper and lower Wiener bounds in THz-TDS measurements. The true value of *n*_eff_ must fall between the extrema defined by the Wiener bounds for any given porosity (Bawuah et al., 2016a). The concept of the Wiener bounds is advantageous in cases where the microstructure of a material is unknown since no assumptions need to be made with regards to the pore shape (Bawuah et al., 2016a; Markl et al., 2018a,a). Further, the Wiener bounds can be applied independent of the sample shape.

Interestingly, the same expressions as in Eqns. 6 and 7 can be derived directly from the AB-EMA model. Assuming a depolarisation factor *L* = 0 (prolate, needle shaped pores) or 1 (flat, oblate pores) results in the upper and lower Wiener bound, respectively (see Fig. 1). It is therefore possible to convert the Wiener bounds into bounds of *L* in cases for which *n*_s_ is unknown. For *f* = 0 the bounds converge to *n*_s_. The best estimate of *n*_s_ in a porous material can therefore be achieved at the lowest porosity where the Wiener bounds are closest together. Eqns. 8 and 9 show these modified expressions of the Wiener bounds resulting in two margins for *n*_s_ based on the sample with lowest porosity (*f*_1_) and its effective refractive index (*n*_eff, 1_). It should be noted that the value of *n*_s_ of a material is a physical constant, similar to the concept of a true density of a material.

(8) $$n_{s,u}^{2}=\frac{1-f_{1}}{\frac{1}{n_{\mathrm{eff},1}^{2}}-f_{1}}\;\text{if}\;L_{1}=1$$

(9) $$n_{s,l}^{2}=\frac{n_{\mathrm{eff},1}^{2}-f_{1}}{1-f_{1}}\;\text{if}\;L_{1}=0$$

Other than *n*_eff_ in the Wiener bounds (Eqns. 6 and 7), the bounds in Eqns. 8 and 9 do not define values that *n*_s_ can take dependent on the pore structure but rather describe the margins it must lie in between. The margins of *n*_s_ can be interpreted as the refractive index that the solid fraction must have if the lowest porosity sample has pores (described by *L*_1_) for which *L*_1_ = 0 or 1 (or in the Wiener bounds formalism: serial or parallel aligned pores) to result in the measured value of *n*_eff, 1_.

Replacing *n*_s_ in Eqns. 3 with each margin results in upper and lower bounds (*L*_u_ and *L*_l_) for *L*:

(10) $$L_{l}=\frac{n_{\mathrm{eff}}^{2}\left(n_{s,l}^{2}-{\mathrm{fn}}_{s,l}^{2}-n_{\mathrm{eff}}^{2}\right)+f}{n_{\mathrm{eff}}^{2}\left(n_{s,l}^{2}-n_{\mathrm{eff}}^{2}+1\right)-n_{s,l}^{2}}$$

(11) $$L_{u}=\frac{n_{\mathrm{eff}}^{2}\left(n_{s,u}^{2}-{\mathrm{fn}}_{s,u}^{2}-n_{\mathrm{eff}}^{2}+f\right)}{n_{\mathrm{eff}}^{2}\left(n_{s,u}^{2}-n_{\mathrm{eff}}^{2}+1\right)-n_{s,u}^{2}}$$

The expressions in Eqns. 8–11 can be used to estimate the values of *n*_s_ and *L* when their true values are unknown.

## 3 Materials and methods

### 3.1 Material

Microcrystalline cellulose (MCC, Avicel PH-102, FMC Europe NV, Brussels, Belgium), lactose *α*-monohydrate (Tablettose 100, Meggle Group, Wasserburg, Germany) and starch (Startab, Colorcon Limited, Dartfort, UK) were used as received.

An immediate release formulation of ibuprofen (BLD Pharmatech, Shanghai, China) was made with dose strength of 10% *w*/*w*. The formulation was composed of common excipients, MCC, lactose anhydrous (Supertab21AN, DFE pharma, Goch, Germany), croscarmellose sodium (CCS, DuPont Nutrition, Wilmington DE, USA) and magnesium stearate (Fisher Scientific, Fair Lawn NJ, USA).

### 3.2 Tableting

The powders were directly compressed with 10 mm round flat-faced and biconvex tooling on a Huxley Bertram 50 compaction simulator (Huxley Bertram Engineering, Cambridge, UK) using a symmetrical compaction profile. The punch depth and radius of curvature of the curved punches for biconvex tablets was 1.05 mm and 12 mm, respectively. The compaction profile was based on a Fette 102i with a rotation speed of 20 rpm corresponding to a die speed of 293mm s^−1^. Each main compaction was proceeded by a pre-compression step with a compression gap of 7.5 mm. The pressure during the main compression was in the range of 5160 MPa. Tablet mass was kept constant at 300 mg, for flat-faced, and 400 mg for biconvex tablets. The material for each tablet was individually weighed, manually filled into the die and compressed.

### 3.3 Porosity determination

The tablet porosity of flat-faced and biconvex tablets was determined based on Eqns. 12 and 13, respectively.

(12) $$f=1-\frac{4M}{\pi D^{2}H\rho_{\text{true}}}$$

(13) $$f=1-\frac{M}{\left[\frac{1}{4}\pi D^{2}\left(H-2h\right)+2\left(\frac{\pi h^{2}}{3}\left(3C-h\right)\right)\right]\rho_{\text{true}}}$$

where *M* is the tablet mass, *D* is the tablet diameter, *H* is the tablet height, *h* is the punch depth of curved punches, *C* is the radius of curvature of curved punches, and *ρ*_true_ is the true density with values of 1.527 kg m^−3^ for the MCC blend and the lactose powder, 1.439 kg m^−3^ for the ibuprofen blend and 1.490 kg m^−3^ for the starch powder.

### 3.4 THz-TDS

THz-TDS measurements were acquired in transmission geometry with the beam propagating through the entire tablet using a Terapulse 4000 spectrometer (Teraview Ltd., Cambridge, UK). The measurement cell was purged with dry nitrogen gas (relative humidity <1%) to avoid any pertubation by water vapour. For each measurement 20 waveforms were averaged. An empty measurement cell was taken as reference and the reference signal was acquired before each tablet measurement.

### 3.5 Data analysis

Data analysis and simulations were carried out using MATLAB 2020a (MathWorks, Natick, MA, USA). Extraction of *n*_eff_ was performed by taking the ratio of the Fourier transform of the time-domain data for each sample and reference pulse. A more detailed description of the extraction from the optical constants from the time-domain can be found in Jepsen and Fischer (2005). For the data analysis *n*_eff_ was averaged in the frequency range of 0.4 – 0.8 THz. The AB-EMA fit was carried out by applying Eq. 3 to the data by varying *L* between 0 and 1 in increments of 0.001. The fit with the smallest standard deviation in *n*_s_ at different porosities was considered best.

### 3.6 Data simulation

Two phase powder compacts of air and solid fraction were simulated based on the AB-EMA (Eq. 3).

#### 3.6.1 Effect of material absorption

To simulate the effect of material absorption on the AB-EMA model samples were simulated with *α*_eff_ ranging from 0 cm^−1^ to 50 cm^−1^ in increments of 1 cm^−1^, *n*_eff_ ranging from 1 to 5 in increments of 0.1 and porosities from 0.05 to 0.25 in increments of 0.05. For each sample *κ*_eff_ was calculated by using the expression $\kappa_{\mathrm{eff}}=\left(c\alpha_{\mathrm{eff}}\right)/\left(4\pi \tilde{\nu }\right)$ where *c* is the speed of light. The frequency $\tilde{\nu }$ was set to 0.2 and 1 THz. Eq. 1 was then used to calculate *n*_s_ with and without accounting for absorption. For the latter case Eq. 1 results in Eq. 3. The pore shape was evaluated based on Eq. 2 with a *p* : *q* ratio of 5, 1 and 1/5 resulting in *L* values of ≈0.053, ≈ 0.33 and ≈0.75.

#### 3.6.2 Variations in the pore shape

To simulate a change in *L* with porosity, a linear model *L*(*f*) = *a*_1_*f* + *a*_2_ was assumed. The simulated porosity ranged from 0.05 to 0.5 in increments of 0.05. The value for *n*_s_ was based on the current in-house estimate of *n*_s_ of the common excipient MCC which is at 1.86. Different sets of tablets were simulated by stepwise changing *L* from 0 to 1 and 1 to 0 for the highest and lowest porosity, respectively in increments of 0.02. Porosities in between were adjusted accordingly. This resulted in the gradient, *a*_1_, taking on values from -2.22 to 2.22 in increments of 0.0889 and resulted in the averaged *L* over all porosities being constant at 0.5.

#### 3.6.3 Density distributions of biconvex tablets

To simulate biconvex tablets with different density distributions, the nominal porosity of the tablets ranged from 0.05 to 0.5 in increments of 0.05. The value for *n*_s_ was 1.86 as described above. The centre porosity was set 10% higher and lower than the nominal porosity to simulate a lower and higher density in the tablet centre. The corresponding *n*_eff_ values were then calculated for the different centre porosities based on Eq. 3 with *L* ≈ 0.33 (spherical pores).

## 4 Results and discussion

### 4.1 Limitations of the AB-EMA

For most materials *n*_s_ cannot be determined directly since a zero-porosity sample is experimentally inaccessible. Instead, *n*_s_ is determined by an extrapolation from the regression analysis of *n*_eff_ over the experimentally accessible porosity range using the AB-EMA (Bawuah et al., 2020).

The correct extraction of *n*_s_ and *L* is of critical importance since they define the AB-EMA fitting model, and therefore the correlation function, used for porosity prediction. Furthermore, knowledge of *n*_s_ is required for more advanced pore shape analyses based on the Wiener bound model (Bawuah et al., 2020). In this study the estimated values of the AB-EMA fit for *n*_s_ and *L* will be referred to as *n*_s, fit_ and *L*_fit_.

#### 4.1.1 Effect of material absorption on the AB-EMA

**Fig. 2:**
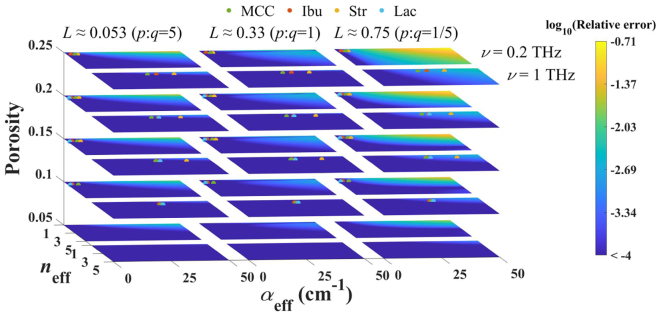
Effect of absorption on the AB-EMA. Relative error of the extraction of *n*_s_ without accounting for absorption as a function of *n*_eff_, *α*_eff_ and porosity at frequencies of 0.2 THz and 1 THz and for *L* values of ≈0.053, ≈0.33 and ≈0.75. The results are depicted for simulated data as well as flat-faced MCC (green, dot), ibuprofen (Ibu, red, dot), starch (Str, yellow, dot) and lactose (Lac, blue, dot) tablets. (For interpretation of the references to colour in this figure legend, the reader is referred to the web version of this article.)

Fig. 2 depicts the relative error in *n*_s_ as a function of porosity, tablet refractive index and absorption coefficient, *α*_eff_, at frequencies of 0.2 THz and 1 THz and for *L* values of ≈0.053, ≈0.33 and ≈0.75. The relative error was calculated as ∣*n*_s, c_ − *n*_s_ ∣ /*n*_s, c_ where *n*_s, c_ and *n*_s_ are the real part of the intrinsic refractive index estimated by applying the AB-EMA with and without accounting for material absorption respectively. The range of *α*_eff_ was based on a non-absorbing material with *α*_eff_ = 0 and a “worst case” scenario in which *α*_eff_ was based on a thin tablet of 1 mm thickness with a transmittance of 1%. The porosity range of 0.05 to 0.25% was based on the typical values used for pharmaceutical tablets. For *n*_eff_, common literature values were considered for setting the range (Lu et al., 2020). The two frequencies investigated are typical limits of the dynamic range of the spectrometer used in our lab when measuring entire tablets in transmission. For *L* Eq. 2 was utilised at a *p* : *q* ratio of 5 (prolate), 1 (spherical) and 1/5 (oblate).

The data shown in Fig. 2 demonstrate that the relative error is highly dependent on *n*_eff_, *α*_eff_, *L*, the porosity and the frequency. However, it should be noted that in any material absorption decreases with increasing porosities. The magnitude of the relative error was found to cover the range of 1 × 10^−0.7^. A general trend towards larger errors at higher absorption and lower values for *n*_eff_ can be observed. However, in some simulated sets intermediate values of *n*_eff_ lead to “islands” of higher errors (e.g. at *L* ≈ 0.33, *f* = 0.25, *ν* = 0.2 THz). An increase in the effect of absorption can be observed at lower frequencies which is due to the frequency dependence of *κ*_eff_ (see Section 2). This should be kept in mind during data analysis since clearly a critical threshold is reached earlier if optical constants are extracted at lower frequencies. The relative error additionally shows a remarkable dependence on the pore shape, described by *L*. Interestingly, under the investigated pore shapes, spherical pores (*L* ≈ 0.33) resulted in the smallest error. For non-spherical a more oblate shape (*L* ≈ 0.75) seems to result in higher errors compared to more prolate pores (*L* ≈ 0.053).

Data extraction of *n*_eff_ typically results in errors on the order of 1 × 10^−4^ to 1 × 10^−3^ (see Supplements Tables 1 to 6). Therefore, in most cases the error caused by the simplified model will be less than that caused by the measurement system itself and the effect due to absorption will be negligible. For comparison, tablets of the typical pharmaceutical excipients MCC, lactose and starch as well as an ibuprofen formulation are shown for which small errors of magnitudes <10^−4^ can be expected. Please note that the pore shape of these tablets was not investigated and therefore the materials are displayed for all three investigated *L* values.

In non-crystalline materials, such as the majority of pharmaceutical excipients, the absorption spectrum exhibits monotonously increasing absorption without any sharp spectral features. In fact, this increase in absorption constitutes the rising flank of the peak due to the vibrational density of states (Sibik et al., 2014). This peak is wider than the spectral bandwidth of any typical THz-TDS instrument that is currently commercially available. Such spectrometers commonly access a range of about 0.1 THz to 3 THz, and in some cases up to about 7 THz, but the dynamic range of all such instruments is decreasing rapidly with frequency and hence for measurements of any tablet sample the usable bandwidth is limited to a maximum of about 2 THz. Therefore, one might expect higher errors at higher frequencies due to the stronger absorption as well as the reduced dynamic range. However, due to the frequency dependency of *κ*_eff_ the effect of increasing absorption is less pronounced. This can also be observed in Fig. 2: although absorption is significantly higher at 1 THz the expected error remains at lower values.

In conclusion, neglecting material absorption during data analysis and application of the AB-EMA is justified in most cases, and in particular for the materials investigated here. The choice of materials used in this study was aimed to represent a wide range of material and structure properties that are typically encountered in pharmaceutical solid dosage forms. In general it is recommended to chose a suitable frequency window for the AB-EMA analysis where the tablet matrix exhibits relatively low absorption. This ensures the validity of the simplified AB-EMA model and further ensures a constant refractive index over a large frequency range in cases where high material absorption is due to a resonance peak in the spectrum. However, extra care must be taken to ensure the assumption is justified when applying the method to materials known to form highly anisotropic pore structures upon compaction as well as with materials with high absorption at low frequencies, as some materials may exhibit values for *α*_eff_ above the considered range. In cases where the validity of these assumptions is uncertain we recommend to extend the AB-EMA model by including the absorption coefficient and using the complex refractive index instead of its real part only. This will effectively eliminate the errors associated with absorption and the AB-EMA model. Since THz-TDS allows for the direct extraction of both parts of the complex refractive index without requiring any additional tools, such as chemometrics or advanced mathematical models such as the Kramers Kronig analysis, this will not affect the experimental setup but only the complexity of the applied model. However, for tablets comprised of such strongly absorbing materials the deterioration of the signal-to-noise-ratio for a measurement in transmission is likely to be a more practical limitation that may prevent application of this method altogether.

#### 4.1.2 Effect of variations in the pore shape on the AB-EMA

To investigate how porosity dependent changes in the pore shape, described by *L* in Eq. 3, affect the accuracy of the AB-EMA fit, powder compacts were simulated as described in Section 3.6. It should be noted that for gradients above 2.04 the algorithm was unable to fit the data. For a gradient of 2.04 the corresponding spheroids have a *p* : *q* ratio of 6.0 and 0.026, at the highest and lowest porosity, respectively (Eq. 2). Such drastic changes in pore shape are unlikely to occur in practice and therefore this was not investigated further in this study.

**Fig. 3:**
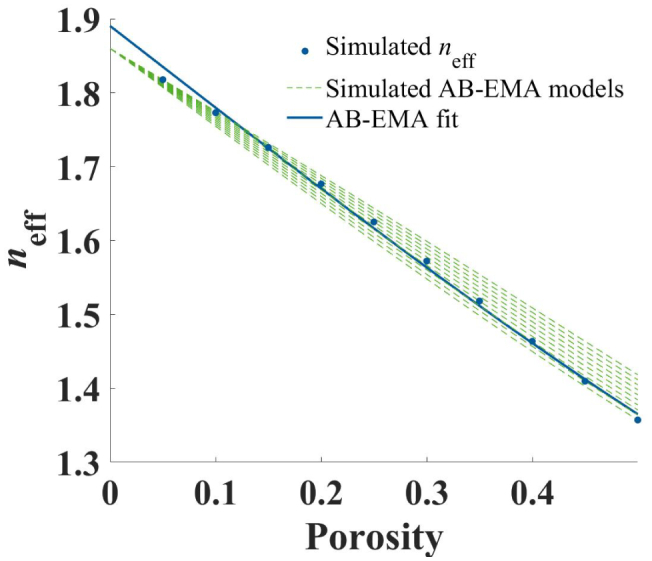
AB-EMA fit of Set 1. Depicted is *n*_eff_ (blue, dot) as a function of porosity, the AB-EMA fit (blue, solid), and the simulated AB-EMA models with varying *L* used in the simulation (green, dashed). (For interpretation of the references to colour in this figure legend, the reader is referred to the web version of this article.)

Fig. 3 illustrates the results for an example set with the simulation parameters, *a*_1_ = 0.5 and *a*_2_ = 0.275 (Set 1). Depicted are the simulated values of *n*_eff_, the different AB-EMA models for each porosity used in the simulation and the AB-EMA fitting function. Just by visual inspection it is clear that the AB-EMA fit was unable to follow the simulated data points and further did not result in a fitting function similar to the simulated AB-EMA models.

**Fig. 4:**
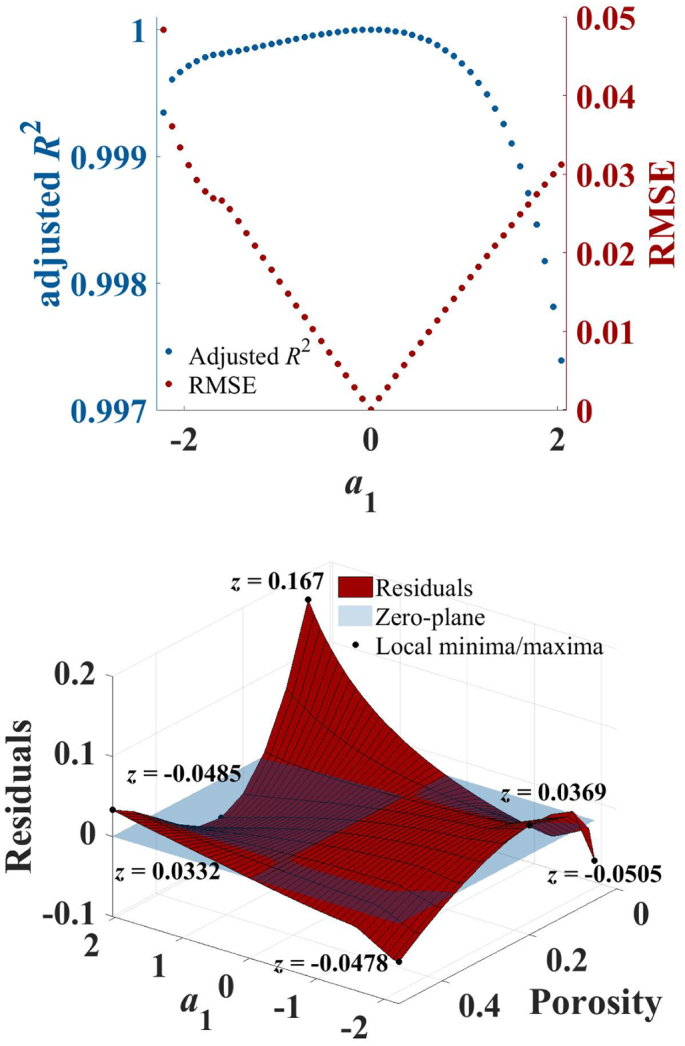
Error analysis of the AB-EMA fit as a function of the gradient. The plot on the top shows the values of the adjusted *R*^2^ (blue, dot) and the RMSE based on porosity (red, dot) as a function of the gradient *a*_1_. The residuals as a function of the gradient and the porosity of the AB-EMA fit are shown on the bottom. (For interpretation of the references to colour in this figure legend, the reader is referred to the web version of this article.)

Fig. 4 illustrates how the adjusted *R*^2^ and the root-mean squared error (RMSE) for the porosity are affected by the gradient (simulation parameter *a*_1_) of *L*. The RMSE was calculated for the independent variable (the porosity) instead of the dependent variable (*n*_eff_). This was considered more practical since for tablet characterisation *n*_eff_ is used to estimate the porosity. For the set with *a*_1_ = 0 both parameters reached a minimum close to zero. This was to be expected since *L* remained constant for this set and could therefore be described by Eq. 3. With higher absolute gradients the fit was impaired gradually. However, the effect on the goodness of fit based on the adjusted *R*^2^ was marginal and even for strong variations in the pore shape the fit was able to describe the data with values above 0.99 at all gradients.

Based on the analysis of the RMSE, deviations in the porosity prediction can be expected to fluctuate by a maximum of around 5% for the theoretically most drastic changes in pore shape. For realistic changes that are more likely to be encountered in tablet processing much lower deviations are to be expected. However, the error was not consistent throughout different porosities as illustrated by the residual surface plot (Fig. 4). Larger residuals were found for the centre porosity and the high and low porosity ends. Therefore, larger deviations between the true and predicted porosity are to be expected at these porosities.

The threshold up to which error a method for porosity prediction is useful will very much depend on the final formulation and the demands on quality and performance of the product. To the best of the authors' knowledge, guidelines currently do not provide specifications regarding tablet porosity. Since extreme gradients in *L* with porosity are unlikely to occur in practice, large deviations in real sets of tablets due to changes in the pore shape are not considered a practical problem. Due to the porosity dependent distribution of the residuals, one should consider an arbitrary fitting model instead of the AB-EMA fit when large deviations in the pore shape can be expected.

**Fig. 5:**
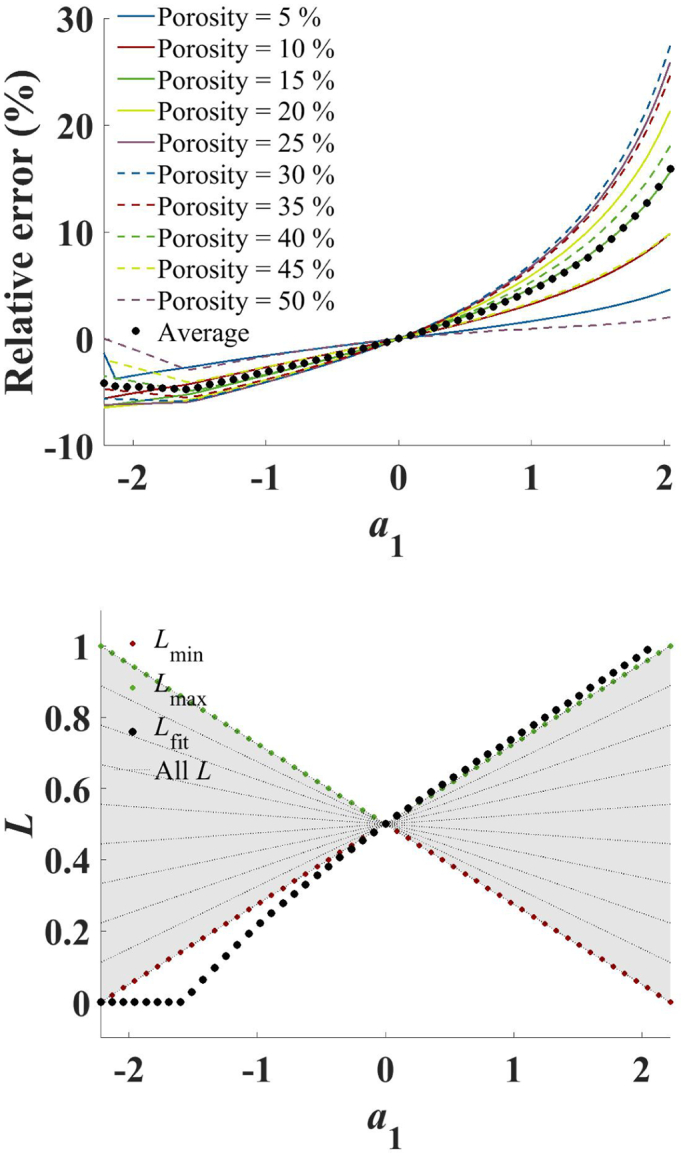
Error analysis of the AB-EMA fitting parameters as a function of the gradient. The relative error of *n*_s, fit_ as a function of the gradient as a mean for all porosities (black, dot) as well as for each individual porosity shown on the top. *L*_fit_ (black, dot), *L*_max_ (green, dot), and *L*_min_ (red, dot) as well as all *L* values in between used in the simulation (grey, dashed) as a function of the gradient (bottom). The grey area illustrates the range of *L* used for each gradient. (For interpretation of the references to colour in this figure legend, the reader is referred to the web version of this article.)

The fitting method for the AB-EMA model selects the value of *L*_fit_ for which applying Eq. 3 results in the least variation of *n*_s, fit_ at the different porosities. The final value of *n*_s, fit_ is then calculated by averaging. Fig. 5 shows a plot of the relative error of *n*_s, fit_ at all porosities, as well as its average, as a function of the gradient *a*_1_. For sets where *L* varied, the gradient strongly affected the accuracy of the fit. For positive gradients an overestimation of *n*_s, fit_ was observed whereas the opposite was the case for negative gradients.

For relatively modest absolute gradients (*m* >  ∣ 0.6∣) relative errors in excess of 2.5%, were determined even without considering any additional experimental errors. In this context, it is interesting to note that the estimates of *n*_s_ for the upper and lower porosity limit were least affected and their relative error remained close to 0. For *L*_fit_ no single reference value existed since a range of values were used in the simulation. *L*_fit_ is considered to be a rough approximation for the description of the pore shape and thus the range of *L* values that were chosen in the simulation (Markl et al., 2018b).

The bottom panel of Fig. 5 shows *L*_fit_ together with the maximum, *L*_max_, and minimum, *L*_min_, *L* values of the simulated data. As in the top panel of Fig. 5, the AB-EMA fit resulted in a perfect estimation of *L* for the zero-gradient set. For most other gradients *L*_fit_ lay outside the range of *L* values used in the simulation (represented by the grey area in Fig. 5). This result is quite unexpected and disproves the aforementioned hypothesis of *L*_fit_ as a rough approximation of *L*. Instead *L*_fit_ did not describe the pore shape of most of the simulated tablet sets since it lay outside the range of *L* values used in most cases. As for the relative error of *L*_fit_, negative and positive gradients resulted in an under- and overestimation, respectively. *L*_fit_ was always closest to the highest porosity sample for which at negative gradients *L*_min_ and for positive gradients *L*_max_ is the corresponding *L* value.

Given these results, it is important to be mindful of the limitations of this approach when adapting the AB-EMA method to data sets where the pore shape, and therefore *L*, cannot be assumed as constant. The tableting process is anisotropic in its nature and deviations in the anisotropy with the pressure can often be expected (Porion et al., 2010). The model of using a linear change in *L* with porosity that was used for this study is only a simplified model for illustrative purposes. In reality more complicated, and indeed formulation and process dependent, deformation behaviour can be assumed. Despite the over simplification of our model the data illustrate that false estimates of *n*_s_ and *L* can be expected even for small changes (i.e. small gradients) in the pore shape upon compaction to different porosities.

Based on the data shown in Fig. 5 one might argue to base the estimate of *L* on the sample with the highest porosity since for this sample the error of *n*_s, fit_ and *L*_fit_ is the smallest. However, this section demonstrated that the AB-EMA model is inadequate to fully describe the pore shape based on THz-TDS measurements and and hence this approach is not recommended.

For porosity prediction the effect of variations in *L* with the pore shape is not considered a critical issue given that the adjusted *R*^2^ values are close to 1 and the RMSE values are very low. Therefore, the AB-EMA is still considered the most suitable approach for porosity prediction. In cases where the AB-EMA fit of a calibration set results in large residuals one can consider the application of other fitting functions which better describe the formulations changes of *n*_eff_ with porosity in order to get a more rigorous porosity estimation. However, a physically meaningful model, such as the ones based on the formalism of the effective medium approximation, will enable us to learn about the formulation mechanistically beyond simple porosity prediction and is therefore more desirable over applying non-physical fitting functions. Nonetheless, our results indicate that a good correlation of the model alone must not be assumed to indicate the correct extraction of *n*_s_ and *L*. A more rigorous method to estimate the applicability of the AB-EMA to a set of data will be introduced in Section 4.2.

#### 4.1.3 Effect of density distribution in biconvex tablets on the pore shape prediction

**Fig. 6:**
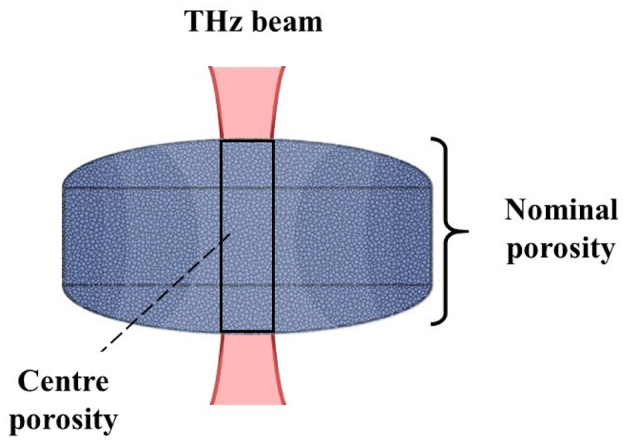
Schematic of the geometry of the beam propagation in a terahertz measurement setup illustrated for a biconvex tablet. The terahertz beam penetrates the centre of the tablet from one face to the other and thereby only probes the centre porosity. In typical setups the beam waist will have dimensions of a couple of millimetres. However, in biconvex tablets, density distribution (illustrated by different shades of blue) might differ from the nominal porosity estimated by tablet weight and dimensions. Please note that this illustration is only accurate for single wavelength beams as the beam width of a Gaussian beam will be frequency dependent. (For interpretation of the references to colour in this figure legend, the reader is referred to the web version of this article.)

Fig. 6 illustrates the THz-TDS setup for biconvex tablets. The terahertz beam propagates through the centre of the tablet from the top to the bottom tablet face. It therefore only measures *n*_eff_ of the centre porosity. In contrast, the nominal porosity of the entire tablet is calculated using the tablet's dimensions and mass and therefore represents the averaged porosity of the sample assuming even distribution of density. In practice the porosity of a sample at its centre is unknown.

To investigate the effect of density distribution on the AB-EMA, tablets were simulated as described in Section 3.6 with *n*_s_ = 1.86 and *L* ≈ 0.33. Even density distribution and a 10% higher and lower centre porosity compared to the nominal porosity were simulated in three different sets. Density distribution was loosely based on the results of the finite element analysis method for biconvex MCC tablets as reported by Sinka et al. (2004), where differences up to 30% between the tablet centre and the nominal porosity were reported. Based on these results the simulated deviation of 10% was considered realistic.

**Fig. 7:**
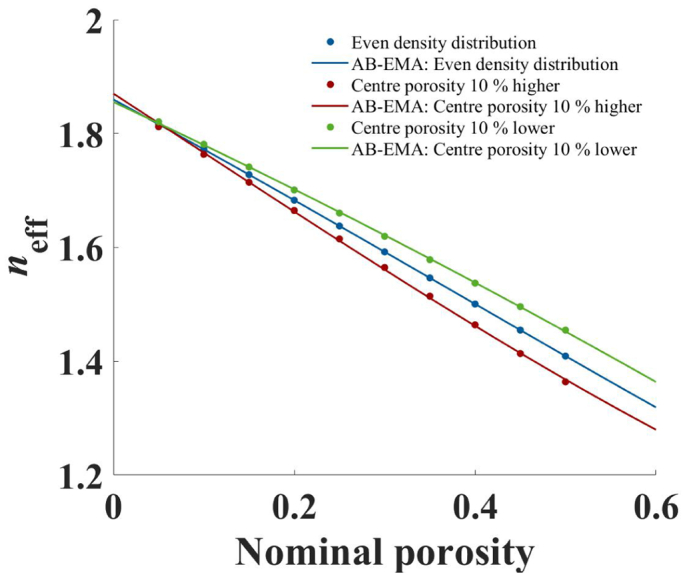
*n*_eff_ and AB-EMA fit as a function of nominal porosity for simulated tablets with density distribution. The results are depicted for tablets with even density distribution (blue) and a centre with 10% higher (red) and lower (green) than average porosity. (For interpretation of the references to colour in this figure legend, the reader is referred to the web version of this article.)

Fig. 7 shows a plot of *n*_eff_ and the AB-EMA fits as a function of nominal porosity. Compared to t he set with even density distribution, *n*_eff_ decreased and increased with higher and lower centre porosity, respectively. This was a consequence of the difference between the nominal porosity and the porosity extracted from the measurement of the tablet centre, which resulted in a corresponding change of *n*_eff_. Naturally, the AB-EMA fit of the evenly distributed sample led to a perfect correlation with the adjusted *R*^2^ being equal to 1. For samples with higher and lower centre porosity the correlation was only marginally impaired with adjusted *R*^2^ values of 0.9993 and 0.9997. *L*_fit_ was found at 0.498 and 0.160 for the sets with higher and lower centre porosity, respectively. Thus, density distribution resulted in a false estimation of *L*, which was fixed to ≈0.33 in all samples. By using Eq. 2, this correspond to a *p* : *q* ratio of approximately 1 : 2 and 2 : 1 for the high and low centre porosity, respectively. Without knowledge of the true cause both could easily be mistaken for plausible pore shapes.

With new insights gained from the analysis of simulated data, non-simulated, flat-faced and biconvex MCC tablets were investigated. The tablet properties are listed in Table 5 and Table 6 in the Supplementary Materials.

**Fig. 8:**
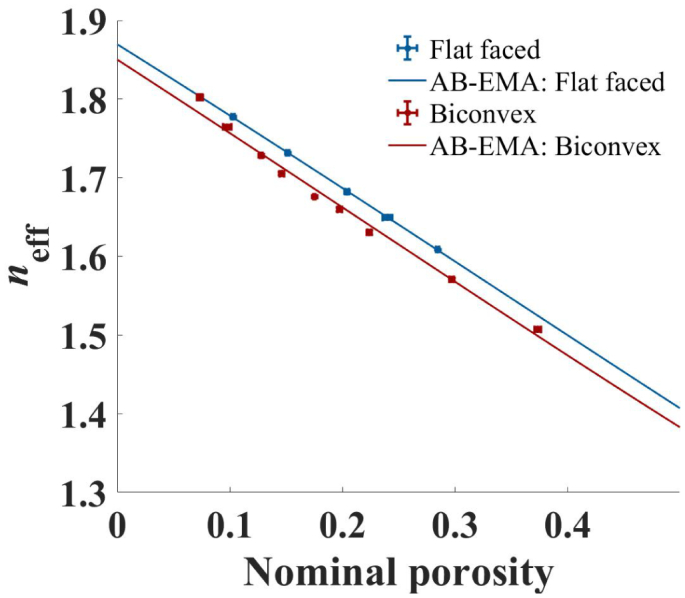
Averaged *n*_eff_ and AB-EMA fit as a function of nominal porosity for flat-faced (blue) and biconvex (red) MCC tablets. Error bars indicate the standard deviation. The data for the individual tablets are shown in the Supplements in Table 5 and Table 6. (For interpretation of the references to colour in this figure legend, the reader is referred to the web version of this article.)

Fig. 8 shows a plot of *n*_eff_ as a function of porosity for the two types of tablets. Averaging in the frequency range of 0.4 – 0.8 THz was performed to account for any small fluctuations in *n*_eff_ as a function of frequency. The frequency range was based on a previously suggested range for porosity measurements of MCC tablets (Bawuah et al., 2020). For the biconvex tablets lower values of *n*_eff_ were observed compared to the flat-faced samples. This suggests a higher centre porosity compared to the nominal porosity. For the AB-EMA fit a good correlation of *n*_eff_ and porosity was found with slightly better results for the flat-faced tablets. Adjusted *R*^2^ values were at 0.9993 and 0.9874 for flat-faced and biconvex tablets, respectively. For the biconvex tablets *L*_fit_ reached a higher value of 0.415 compared to 0.357 for flat-faced tablets.

Based on the previously gained results for the simulated data it is suggested that the higher value of *L*_fit_ in the biconvex tablets originated from density distribution in this set. In this case an assumption of equal centre and nominal porosity would result in an apparent change in the pore shape compared to flat-faced tablets when using the AB-EMA. Based on the simulation, the higher value of *L*_fit_ corresponds to a higher centre porosity. This is well in line with the lower values found for *n*_eff_ in this set compared to the flat-faced tablets.

Several studies suggest that biconvex tablets often show higher porosity in the centre compared to the edges (Sinka et al., 2004; May et al., 2013; Diarra et al., 2015). In contrast, flat-faced tablets are not expected to show significant density distribution in the radial direction, if any (Diarra et al., 2015). Therefore, the set of flat-faced tablets can be used for the accurate extraction of the material property, *n*_s_, and thus to reliably predict the centre porosity of biconvex tablets.

It should be noted that for both, biconvex and flat-faced tablets, density distribution in the axial direction, that is the direction the force is applied in during compaction, may very well be present. The porosity obtained by THz-TDS is always an average measurement that is not able to resolve any axial density distribution. It is well-known that the vast majority of axial density anisotropy is removed during elastic recovery in the unloading step within the die of the tablet press and the effects due to any residual remaining strain in the tablet is the subject of current research.

Based on the terahertz analysis, an average ratio between the porosity measured at the tablet centre and the nominal porosity of 0.875 was found for the biconvex tablets investigated here. As already concluded from the lower *n*_eff_ values for biconvex tablets, the centre porosity was therefore higher compared to the nominal porosity. Considering the similar density distribution in the real tablets and the simulated tablets with lower centre porosity one would expect a higher deviation in *L*_fit_ between the biconvex and flat-faced tablets. This could be rooted in the high variation of density distribution between tablets of different porosities (See Supplements Fig.12). Taking this into account the experimental and simulated deviations in *L*_fit_ are considered to be in good agreement by the authors.

Data analysis of simulated data demonstrated that density distribution in biconvex tablets highly affects the AB-EMA methodology when the nominal porosity of the tablet is considered equal to the centre porosity. In this case the AB-EMA fit results in an inaccurate estimation of *L*_fit_. Similar as the variation in pore shape discussed in Section 4.1.2, the goodness of fit was not impaired significantly by density distribution. This again creates a situation where one might not be able to establish whether the extracted parameters are accurate based on the ability of the AB-EMA to correlate *n*_eff_ and porosity. Previously, a change in pore shape with tablet geometry was reported by applying the AB-EMA on THz-TDS measurements (Bawuah et al., 2019). Based on the results discussed in this section it is suggested that this may be rooted in the density distribution of biconvex tablets rather than an actual change in pore shape. This is consistent with the density distribution suggested for the here reported biconvex MCC tablets.

In conclusion, it is recommended to perform all calibration measurements using flat-faced tablets rather than biconvex or other shapes that may result in an anisotropic density distribution. The results obtained from the flat-faced tablets can then be directly used together with the AB-EMA method to accurately measure the centre porosity of biconvex tablets, a metric which cannot be determined easily by other methods. The tablet centre porosity may in many cases constitute a key critical quality attribute of an immediate release tablet with regards to disintegration due to its commonly higher porosity compared to the rest of the tablet matrix.

### 4.2 Extensions of the AB-EMA model to describe variations in the pore shape

Section 4.1.2 demonstrated how tablet anisotropy affects the AB-EMA and the extraction of *n*_s_ and *L*. Although the AB-EMA resulted in a strong correlation, it was unable to extract the true physical properties of the material when pore shape variations or density distribution were simulated. Therefore, the accuracy of the extracted parameters cannot be assessed based on the goodness of fit alone. This section discusses alternative models to evaluate the applicability of the AB-EMA fit and to investigate *L* as a function of porosity.

#### 4.2.1 Simulated data

**Fig. 9:**
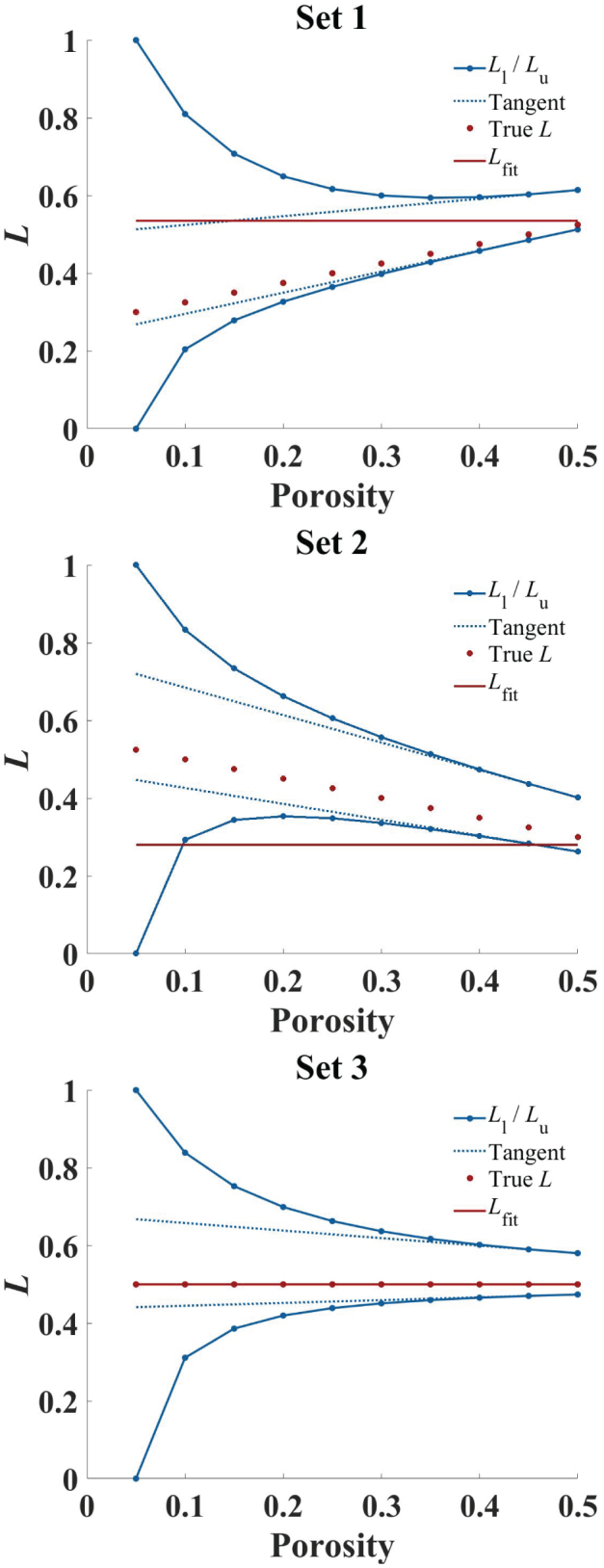
*L*_l_ and *L*_u_ for simulated sets with varying gradient of *L* as a function of porosity. Depicted are *L*_l_ and *L*_u_ (blue, dot), the tangents based on the two highest porosities (blue, dashed), true *L* (red, dot), and *L*_fit_ (red, solid) as a function of porosity for Set 1 (a), Set 2 (b), and Set 3 (c). (For interpretation of the references to colour in this figure legend, the reader is referred to the web version of this article.)

To evaluate the new approach based on the concept of Wiener bounds, three sets of simulated data were used with corresponding linear models of *a*_1_ = 0.5 and *a*_2_ = 0.275 (Set 1), *a*_1_ =  − 0.5 and *a*_2_ = 0.55 (Set 2), and *a*_1_ = 0 and *a*_2_ = 0.5 (Set 3). Fig. 9 depicts *L*_l_ and *L*_u_ for the three sets. The bounds were chosen based on the sample with lowest porosity and thus information got lost for this sample and the upper and lower bound resulted in values of 0 and 1 by default.

With increasing porosity, the bounds approach each other and their tangents ultimately converge parallel to one another and the simulated gradient of *L*. For Set 3 this results in a convergence towards a constant value of *L*, whereas for Sets 1 and 2 a close to linear in- and decrease, respectively, towards the high porosity end can be observed.

Fig. 9 also depicts the values of *L*_fit_ of the AB-EMA fit for the three sets. In Section 4.1.2 it was discussed that variations in the pore shape can result in a false estimate of *L*_fit_. This can also be seen when comparing *L*_fit_ with the bound progression in Sets 1 and 2. There is a clear deviation between the constant progression in *L*_fit_ and the gradient for the bounds at higher porosities. Furthermore, for Set 2 *L*_fit_ remained below the bounds for porosities between 0.1 and 0.45. This is not possible since the bounds display the two extremes in which *L* must lie in.

Therefore, the bounds can be used as a simple method to validate the AB-EMA fit. If the bounds converge towards a steep gradient, or if *L*_fit_ lies outside the calculated bounds at certain porosities, the AB-EMA fit is invalid and other methods need to be applied. No prior knowledge about the deformation behaviour of the material needs to be obtained. The THz-TDS data can be directly used to estimate the validity of the AB-EMA fit to avoid errors associated with a change in the pore shape (as discussed in Section 4.1.2) and to chose alternative models for a better description of the investigated tablet set, one of which will be introduced in Section 4.2.2. Furthermore, for high porosities a good approximation of the pore shape can be made due to the proximity of the bounds. Lastly, bounds of multiple sets with different pore shapes can be used to limit the range of possible *L* values.

#### 4.2.2 Comparison of different size fractions of MCC applying the bounds for L

Skelbæk-Pedersen et al. (2020a) previously investigated the effect of fragmentation during tableting on the water ingress kinetics using terahertz pulsed imaging. Their data was reused in this section to evaluate the newly introduced concept of upper and lower bounds for *L* and the effect of deformation on the AB-EMA on non-simulated tablets. Two sets of tablets compacted with different MCC particle size fractions in the range of <125 μm and 355 μm to 500 μm were analysed by THz-TDS. The materials were compressed into round, flat-faced tablets at different pressures resulting in porosities of 0.05 to 0.3. The powder mass was adjusted accordingly to retain a constant tablet thickness. For further discussion, the two powders will be referred to as small and large size fractions for particle sizes of <125 μm and 355 μm to 500 μm, respectively.

**Fig. 10:**
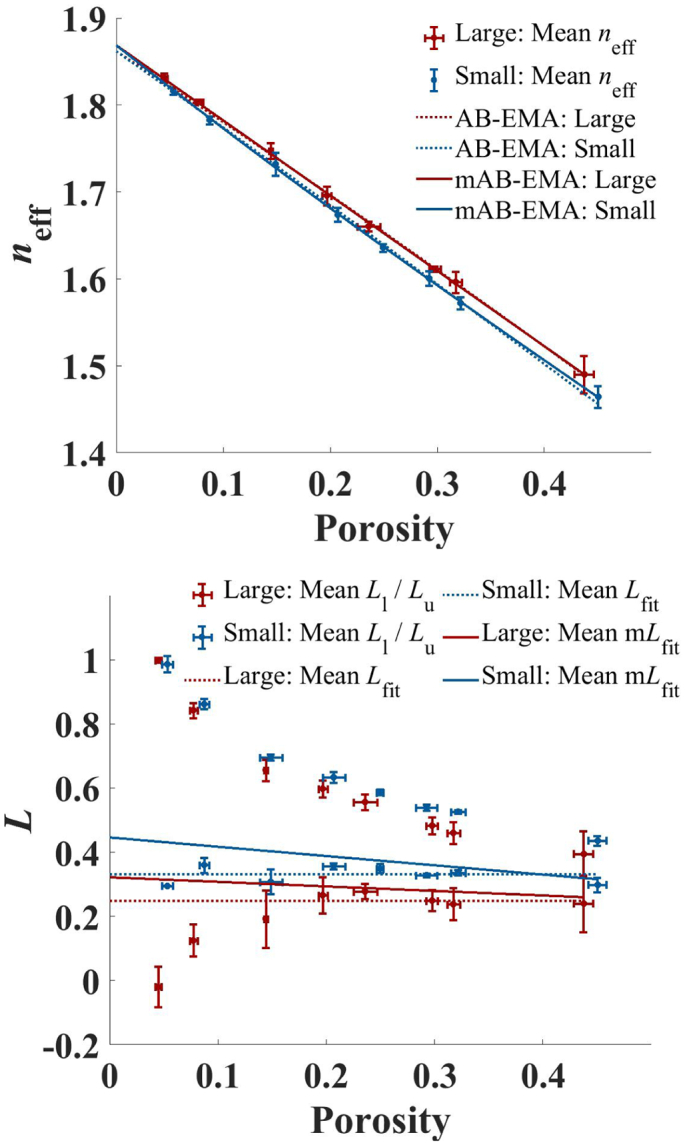
THz-TDS based porosity analysis of MCC tablets of different size fractions. Depicted is *n*_eff_ (dot), the AB-EMA (dashed), and the mAB-EMA (solid) fit as a function of porosity (left) as well as *L*_l_/*L*_u_ (dot) and *L*_fit_ based on the AB-EMA (dashed) and the mAB-EMA (solid) model as a function of porosity (right) for two sets of MCC tablets with initial particle sizes of <125 μm (blue) and 355 μm to 500 μm (red). Error bars indicate the standard deviation. (For interpretation of the references to colour in this figure legend, the reader is referred to the web version of this article.)

Fig. 10 depicts *n*_eff_ and the corresponding AB-EMA fit as a function of porosity for tablets of both size fractions. For the larger size fraction higher values of *n*_eff_ were measured. Both size fractions are composed of the same material and therefore, *n*_s_ must not change between size fractions. The fitting method was adjusted to result in the best model for which *n*_s, fit_ takes on the same value for both size fractions. The AB-EMA fit estimated *n*_s, fit_ = 1.867 and *L*_fit_ at 0.259 and 0.339 respectively for the large and small size fraction.

This means that the AB-EMA predicted more oblate shaped pores for tablets of the small compared to the large size fraction. To evaluate the accuracy of the fit, *L*_l_, *L*_u_, and *L*_fit_ are plotted as a function of porosity in the bottom panel of Fig. 10. Measurements of tablets samples prepared from the small size fraction powder resulted in higher values of *L*_l_ and *L*_u_. Thus, similar as for *L*_fit_ the bounds suggest a more oblate pore shape for the small size fraction. The values of *L*_fit_ fell outside the bounds for porosities between 0.20 – 0.30 for the large size fraction and porosities at 0.10; 0.20; 0.25; 0.33 for the small size fraction.

For these samples the extracted values of *L*_fit_ must be erroneous and the AB-EMA model thus failed to describe the data. The progression of the bounds at high porosities suggests a negative gradient of *L* for both tablet sets. Such a change in *L* would likely result from an incorrect estimate of *L* by the AB-EMA fit as discussed in Section 4.1.2. Furthermore, based on Fig. 4 the negative gradient should result in an underestimate of *L*_fit_ compared to the range of true *L* values of the pores. This is in accordance with values of *L*_fit_ falling below the bounds at some porosities for the two types of tablets. It can therefore be concluded that the values of *L*_fit_ must indeed be underestimated at these porosities. The results suggest that the AB-EMA method is inadequate to describe the two sets of tablets because of pore shape changes in these tablets under the conditions studied.

To adjust the AB-EMA and allow for changes in the pore structure with the porosity to be accommodated, the *L* term can be replaced by a porosity dependent expression. It was found that replacing *L* in Eq. 3 with a simple linear model of the form *L*(*f*) = *a*_1_*f* + *a*_2_ can describe the data well. It is helpful to highlight that replacing *L* with this model still allows for a constant value of *L* in cases where the gradient, *a*_1_ = 0. The modified AB-EMA (mAB-EMA) model is shown in Fig. 10 as a function of porosity. The mAB-EMA model estimated higher values of *n*_s_ at 1.869. It resulted in slightly higher adjusted *R*^2^ values of 0.9942 and 0.9991 for the large and small size fraction compared to the AB-EMA (adjusted *R*^2^ at 0.9936 and 0.9984). *L*_fit_ of the mAB-EMA lay inside the bounds over the entire range of porosities (Fig. 10). The model resulted in a clear gradient of −0.141 and  ‐− 0.289 of *L*_fit_ as a function of porosity for the large and small size fraction, respectively. This further supports a change in pore shape towards more oblate pores at lower porosities as already suggested by the progression of the bounds. Additionally, it resulted in larger values of *L*_fit_ for the smaller size fraction and therefore suggests more oblate pores compared to the large size fraction, in line with the other models.

Without any further data processing the data shown in Fig. 10 already suggest a difference in tablet microstructure in the two size fractions based on the different values of *n*_eff_ at a specific porosity. For tablets of the same material with the same microstructure *n*_eff_ should result in the same value. However, while the work by SkelbÃ¦k-Pedersen et al. did report a difference in microstructure between tablets of different MCC size fractions (Skelbæk-Pedersen et al., 2020a, Skelbæk-Pedersen et al., 2020b, Skelbæk-Pedersen et al., 2020c), in their study on measuring water ingress into the porous tablet matrix it was not explored specifically that THz-TDS is able to measure this difference in structure but the work focused on the liquid transport kinetics (Skelbæk-Pedersen et al., 2020a). Further analysis of the data show that the AB-EMA model, *L*_l_ and *L*_u_, and the mAB-EMA both suggest more oblate shaped pores, based on higher *L* values, for the tablets prepared from the small compared to the large size fraction.

It was furthermore reported that an increase in particle size results in a higher fragmentation degree during tableting (Skelbæk-Pedersen et al., 2020c). Small size fractions are therefore expected to show less fragmentation. This was demonstrated on the same size fractions of MCC as used in this study. If less of the energy during compaction is converted into particle fragmentation, plastic deformation must occur to a higher degree for tablets of the same final porosity given the amount of elastic deformation is materials dependent and therefore constant. For MCC it is well-known that plastic deformation is the major deformation route (Skelbæk-Pedersen et al., 2020c) and therefore tableting might result in a higher degree of plastic deformation in smaller size fractions due to a lesser degree of fragmentation.

Such behaviour would be in line with the pore shape analysis presented in this section. The higher degree of plastic deformation would result in relatively more flatter particles in the resulting tablets. Since pore shape is considered the indirect result of the shape of the surrounding particles this would in turn result in a flattening of pore shape as well. Wu et al. (2008) reported the same for cubic starch compacts based on image analysis methodology. Upon compression particle deformation resulted in “pancake” like structures. Pores were formed by stacking of the particles and in turn exhibited high anisotropy (Wu et al., 2008).

In the AB-EMA model this would correspond to more oblate pores with *L* values closer to 1 as observed in this study. An effect of plastic deformation on *L* can be further suggested based on the negative gradient of *L*_fit_ obtained by the mAB-EMA as well as indicated by *L*_l_ and *L*_u_. This gradient is likely to be the result of particle deformation upon higher compression pressures. Therefore, both models, the bounds for *L* and the mAB-EMA, predicted a change in the pore shape well in line with theoretical considerations regarding plastic deformation of the two tablet types.

#### 4.2.3 Effect of density distribution on the bounds for L

**Fig. 11:**
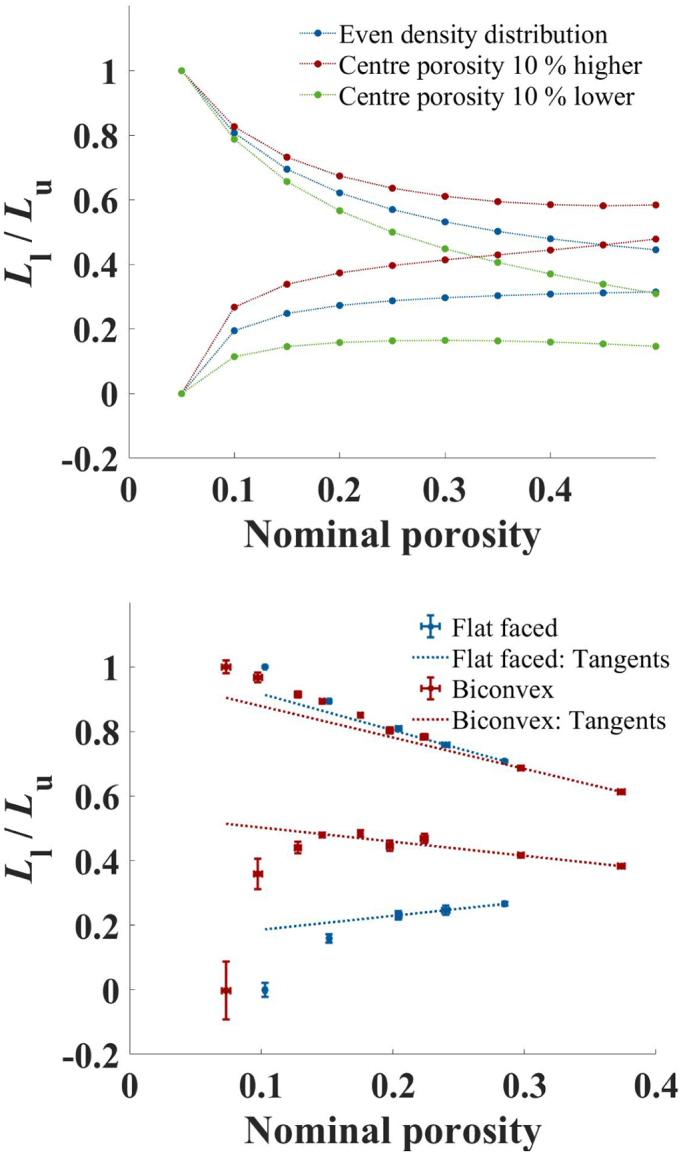
Evaluation of *L*_l_ and *L*_u_ of biconvex tablets. The bounds are depicted for simulated biconvex tablets without density distribution (blue) and a higher (red) and lower (green) centre porosity (left) as well as for real flat-faced (blue) and biconvex (red) MCC tablets (right). (For interpretation of the references to colour in this figure legend, the reader is referred to the web version of this article.)

In Section 4.1.3 the effect of density distribution in biconvex tablets on the AB-EMA method was discussed. The same sets of simulated and real biconvex and flat-faced tablets are re-analysed for this section. To evaluate the effect of density distribution on the bounds, *L*_l_ and *L*_u_, of the simulated tablets were plotted as a function of porosity in Fig. 11.

For the three sets the bounds progressed differently. This suggests a difference in the porosity dependent pore shape. However, *L* was kept constant at ≈0.33 in the simulation. The difference in the observed trend is therefore purely the result of density distribution and the false assumption of equal centre and nominal porosity. Here higher centre porosities (as expected for most biconvex tablets, (Sinka et al., 2004; May et al., 2013; Diarra et al., 2015)) resulted in a strong apparent dependency towards more oblate pores at lower porosities.

Fig. 11 illustrates the same analysis on non-simulated biconvex and flat-faced MCC tablets. The porosity range of the two tablet sets, while being similar, was not the same. This resulted in the bounds for the flat-faced tablets being further apart since in this set the lower end of the porosity range was higher compared to the biconvex tablets. For the flat-faced tablets, no clear trend in *L*_l_ and *L*_u_ as a function of porosity was observed. For the biconvex tablets, *L*_l_ and *L*_u_ indicate a strong trend towards higher values at lower porosities. Based on this, one would expect the AB-EMA to be unable to represent the data, which is consistent with the results in Section 4.1.3.

Since for biconvex tablets density distribution is common, one should keep this in mind when attributing the trend in *L*_l_ and *L*_u_ to a change in pore shape. Based on the results for the simulated data it is suggested that the strong dependence is in fact the result of the higher centre porosity estimated in Section 4.1.3. This itself is not necessarily a disadvantage when *L*_l_ and *L*_u_ are used to test the applicability of the AB-EMA fit. Steep gradients of the bounds at higher porosities indicate that the AB-EMA cannot be applied for the analysed set. It does not matter whether this is due to density distribution or pore shape changes. Furthermore, it can be useful when investigating density distribution in biconvex tablets with THz-TDS. To differentiate between the effect of density distribution and pore shape on *L*_l_ and *L*_u_ the results between a flat-faced calibration set and the biconvex analysis set can be used. As demonstrated in this section a difference in *n*_eff_ of flat-faced and biconvex tablets at the same nominal porosity indicates density distribution in the radial direction. When the centre porosity is expected to differ from the nominal porosity, *L*_l_ and *L*_u_ should not be used to investigate the pore shape of biconvex tablets.

## 5 Conclusion

Different error sources of the AB-EMA for the extraction of porosity and pore shape of pharmaceutical tablets with THz-TDS were investigated. Absorption was found to have a negligible impact on the accuracy of the model. Pore shape changes with porosity and density distribution did both not significantly impair the quality of the AB-EMA fit for porosity estimation of pharmaceutical tablets. However, density distribution and pore shape changes highly influenced the accuracy of the estimation of *n*_s_ and *L*. Further, the only marginal impairment of the goodness of fit increases the danger of misinterpreting the extracted parameters.

An alternative model was suggested to convert the Wiener bounds into bounds of *L*. It was demonstrated that the model can be used to evaluate i) whether changes in the pore shape with porosity occur and ii) the applicability of the AB-EMA fit. Furthermore, a modified AB-EMA (mAB-EMA) model was introduced allowing for a linear change in *L* with porosity. The mAB-EMA model was found useful as a correlation function as well as for pore shape analysis.

Lastly, density distribution was found to affect the newly introduced bound model for *L*. However, it was suggested that for such cases the model is still useful for evaluating density distribution although the physical description of pore shape is lost.

## Declaration of Competing Interest

The authors declare that they have no known competing financial interests or personal relationships that could have appeared to influence the work reported in this paper.

## Appendix A Supplementary data

Supplementary material Tabulated physical parameters of the samples used in the present study (diameter, thickness, weight, porosity and effective refractive index). Supplementary material
