# Supplementary material for: Terahertz time-domain spectroscopy for powder compact porosity and pore shape measurements: An error analysis of the anisotropic bruggeman model
Source: Int J Pharm X. 2021 Apr 27;3:100079. doi: 10.1016/j.ijpx.2021.100079 (PMC8120941; doi:10.1016/j.ijpx.2021.100079)
Supplement: Supplementary material — Tabulated physical parameters of the samples used in the present study (diameter, thickness, weight, porosity and effective refractive index). [file mmc1.pdf]

Table 1: flat-faced MCC tablets. Physical dimensions and characteristics, including $\sigma(n_{\text{eff}})$ and $\sigma(\alpha_{\text{eff}})$, the standard deviation of n_{eff} and α_{eff} in the frequency range of 0.4 – 0.8 THz.

Diameter (mm)	Thickness (mm)	Weight (mg)	Porosity (-)	n_{eff} (-)	$\sigma(n_{\text{eff}})$ ($\times10^{-4}$)	α_{eff} (cm^{-1})	$\sigma(\alpha_{\text{eff}})$ ($\times10^{-2}\,\text{cm}^{-1}$)
10.016	2.980	298.5	0.1468	1.7449	11.711	39.384	2.6432
10.017	2.960	293.7	0.1549	1.7377	11.741	39.5688	2.6736
10.017	2.958	294.5	0.1521	1.7416	11.775	39.034	2.6392
10.038	3.158	297.1	0.2021	1.6908	10.708	37.8871	2.3994
10.031	3.162	295.3	0.2069	1.6858	10.663	36.6482	2.3180
10.037	3.148	291.8	0.2137	1.6800	10.673	36.1102	2.2942
10.043	3.377	294.5	0.2612	1.6299	9.6530	33.8056	2.0021
10.044	3.354	289.6	0.2686	1.6244	9.6861	35.1908	2.0984
10.045	3.375	293.7	0.2630	1.6279	9.6466	33.6477	1.9939

Table 2: Diameter, thickness, weight, porosity, n_{eff} and the standard deviation of n_{eff} (0.4 – 0.8 THz) of flat-faced Ibuprofen tablets.

Diameter (mm)	Thickness (mm)	Weight (mg)	Porosity (-)	n_{eff} (-)	$\sigma(n_{\text{eff}})$ ($\times10^{-4}$)	α_{eff} (cm^{-1})	$\sigma(\alpha_{\text{eff}})$ ($\times10^{-2}\,\text{cm}^{-1}$)
10.041	2.790	300.3	0.1098	1.7858	12.802	28.691	2.0567
10.045	2.791	299.2	0.1141	1.7836	12.781	29.345	2.1028
10.045	2.796	301.7	0.1083	1.7860	12.776	29.211	2.0895
10.045	2.922	299.4	0.1532	1.7466	11.955	27.967	1.9143
10.036	2.922	300.0	0.1501	1.7470	11.958	27.705	1.8963
10.044	2.924	300.7	0.1500	1.7437	11.927	28.087	1.9211
10.064	3.119	300.0	0.2082	1.6965	10.879	27.387	1.7561
10.025	3.114	300.4	0.1996	1.6967	10.898	27.791	1.7849
10.025	3.117	299.5	0.2028	1.6952	10.877	27.339	1.7542

Table 3: Diameter, thickness, weight, porosity, n_{eff} and the standard deviation of n_{eff} (0.4 – 0.8 THz) of flat-faced lactose tablets.

Diameter (mm)	Thickness (mm)	Weight (mg)	Porosity (-)	n_{eff} (-)	$\sigma(n_{\text{eff}})$ ($\times 10^{-4}$)	α_{eff} (cm^{-1})	$\sigma(\alpha_{\text{eff}})$ ($\times 10^{-2}\,\text{cm}^{-1}$)
10.04	2.952	298.6	0.1121	1.7316	11.732	28.105	1.9042
10.041	2.951	296.8	0.1174	1.7207	11.662	26.613	1.8037
10.037	2.956	300.3	0.1078	1.7248	11.670	27.796	1.8807
10.046	3.121	300.1	0.1570	1.6830	10.786	26.117	1.6736
10.046	3.115	299.6	0.1568	1.6870	10.832	26.771	1.7188
10.042	3.121	299.7	0.1575	1.6846	10.795	27.078	1.7352
10.046	3.307	299.6	0.2058	1.6419	9.9300	28.990	1.7533
10.042	3.312	299.9	0.2056	1.6382	9.8930	26.086	1.5752
10.045	3.310	299.5	0.2066	1.6406	9.9129	26.917	1.6264
10.045	3.519	299.5	0.2537	1.6078	9.1376	26.619	1.5129
10.051	3.512	299.4	0.2534	1.5995	9.1088	26.820	1.5274
10.049	3.520	300.4	0.2523	1.5995	9.0881	26.340	1.4966

Table 4: Diameter, thickness, weight, porosity, n_{eff} and the standard deviation of n_{eff} (0.4 – 0.8 THz) of flat-faced starch tablets.

Diameter (mm)	Thickness (mm)	Weight (mg)	Porosity (-)	n_{eff} (-)	$\sigma(n_{\mathrm{eff}})$ ($\times 10^{-4}$)	α_{eff} (cm^{-1})	$\sigma(\alpha_{\mathrm{eff}})$ ($\times 10^{-2}\,\mathrm{cm}^{-1}$)
10.004	2.767	302.0	0.0907	1.7734	12.818	28.504	2.0603
10.009	2.766	300.7	0.9516	1.7694	12.794	28.452	2.0573
10.008	2.762	297.9	0.1021	1.7630	12.766	27.519	1.9927
10.016	2.922	299.4	0.1484	1.7220	11.787	25.966	1.7773
10.011	2.923	299.5	0.1475	1.7218	11.781	25.929	1.7741
10.011	2.921	301.4	0.1415	1.7250	11.811	26.132	1.7893
10.021	3.110	300.9	0.1966	1.6765	10.782	24.383	1.5680
10.022	3.110	298.8	0.2024	1.6723	10.755	24.276	1.5612
10.017	3.107	300.6	0.1960	1.6764	10.791	24.280	1.5629
10.026	3.307	300.7	0.2458	1.6337	9.8800	22.751	1.3759
10.025	3.313	302.1	0.2434	1.6335	9.8610	22.867	1.3804
10.022	3.309	300.0	0.2473	1.6317	9.8621	22.495	1.3596

Table 5: Diameter, thickness, weight, porosity, n_{eff} and the standard deviation of n_{eff} (0.4 – 0.8 THz) of biconvex MCC tablets.

Diameter (mm)	Thickness (mm)	Weight (mg)	Porosity (-)	n_{eff} (-)	$\sigma(n_{\mathrm{eff}})$ ($\times 10^{-4}$)
10.014	3.211	300.3	0.1033	1.7760	11.828
10.017	3.226	301.8	0.1041	1.7764	11.771
10.014	3.218	300.7	0.1025	1.7767	11.817
10.009	3.223	301.0	0.1011	1.7802	11.828
10.019	3.218	300.5	0.1028	1.7772	11.828
10.014	3.237	302.7	0.1032	1.7773	11.742
10.023	3.232	318.3	0.150	1.7340	10.895
10.030	3.213	317.0	0.1527	1.7308	10.920
10.022	3.215	317.8	0.153	1.7313	10.895
10.023	3.213	316.9	0.1513	1.7306	10.922
10.020	3.217	317.7	0.1518	1.7299	10.890
10.019	3.227	318.4	0.1509	1.7325	10.882
10.038	3.206	337.2	0.2065	1.6818	9.9748
10.037	3.188	335.4	0.2065	1.6808	10.022
10.034	3.195	335.2	0.2038	1.6823	10.037
10.027	3.209	336.0	0.2011	1.6820	10.011
10.036	3.228	338.2	0.2031	1.6846	9.9619
10.036	3.205	336.3	0.2043	1.6805	9.9941
10.044	3.205	351.4	0.2397	1.6513	9.3982
10.031	3.209	352.4	0.2389	1.6495	9.3617
10.049	3.203	353.7	0.2458	1.6425	9.2878
10.043	3.206	350.7	0.2378	1.6549	9.4380
10.040	3.213	352.4	0.2393	1.6484	9.3553
10.039	3.212	352.6	0.2399	1.6512	9.3658
10.044	3.214	375.2	0.2859	1.6081	8.5719
10.049	3.205	373.4	0.2852	1.6081	8.6133
10.048	3.211	373.5	0.2839	1.6111	8.6270
10.049	3.218	375.0	0.2854	1.6078	8.5747
10.043	3.218	375.0	0.2845	1.6079	8.5756
10.043	3.219	374.9	0.2849	1.6080	8.5756

Table 6: Diameter, thickness, weight, porosity, n_{eff} and the standard deviation of n_{eff} (0.4 – 0.8 THz) of biconvex MCC tablets.

Diameter (mm)	Thickness (mm)	Weight (mg)	Porosity (-)	n_{eff} (-)	$\sigma(n_{\mathrm{eff}})$ ($\times 10^{-4}$)
10.008	3.247	398.2	0.0625	1.8021	9.0512
10.007	3.289	401.0	0.0593	1.8026	8.9904
10.009	3.274	400.4	0.0619	1.7976	8.9791
10.005	3.315	402.1	0.0551	1.8028	8.9670
10.006	3.283	400.9	0.0605	1.8035	8.9972
10.004	3.274	399.6	0.0587	1.8049	9.0335
10.007	3.290	410.3	0.0879	1.7595	8.5764
10.004	3.278	407.4	0.0820	1.7671	8.6749
10.007	3.298	409.7	0.0838	1.7671	8.6262
10.007	3.290	410.5	0.0885	1.7598	8.5738
10.011	3.298	411.1	0.0885	1.7667	8.5948
10.009	3.330	413.9	0.0879	1.7660	8.5334
10.011	3.272	420.0	0.1215	1.7269	8.2233
10.014	3.274	420.1	0.1216	1.7283	8.2280
10.010	3.294	421.4	0.1194	1.7277	8.1998
10.015	3.282	420.8	0.1215	1.7270	8.2081
10.011	3.290	420.9	0.1192	1.7295	8.2182
10.012	3.270	419.5	0.1207	1.7299	8.2475
10.020	3.291	428.2	0.1400	1.7077	7.9761
10.020	3.296	429.5	0.1422	1.7036	7.9329
10.016	3.286	428.0	0.1403	1.7068	7.9756
10.018	3.296	428.4	0.1390	1.7069	7.9689
10.017	3.291	429.2	0.1423	1.7025	7.9333
10.021	3.281	427.8	0.1417	1.7046	7.9692
10.022	3.276	439.3	0.1728	1.6756	7.6284
10.019	3.297	440.6	0.1704	1.6756	7.6061
10.019	3.283	439.3	0.1707	1.6762	7.6310
10.019	3.275	439.3	0.1727	1.6747	7.6243
10.019	3.287	440.1	0.1717	1.6770	7.6211
10.020	3.276	439.5	0.1731	1.6751	7.6228
10.029	3.288	447.9	0.1916	1.6587	7.4065
10.028	3.305	449.8	0.1918	1.6611	7.3861
10.029	3.285	448.2	0.1930	1.6627	7.4196
10.032	3.257	447.9	0.1995	1.6582	7.4042
10.032	3.282	449.5	0.1972	1.6621	7.3955
10.031	3.299	450.8	0.1959	1.6579	7.3552
10.028	3.295	460.0	0.2176	1.6310	7.0915
10.032	3.269	459.2	0.2224	1.6314	7.1054
10.030	3.278	460.3	0.2225	1.6291	7.0786
10.034	3.278	460.2	0.2227	1.6327	7.0958
10.036	3.267	458.5	0.2218	1.6285	7.1036
10.035	3.288	460.9	0.2220	1.6295	7.0709

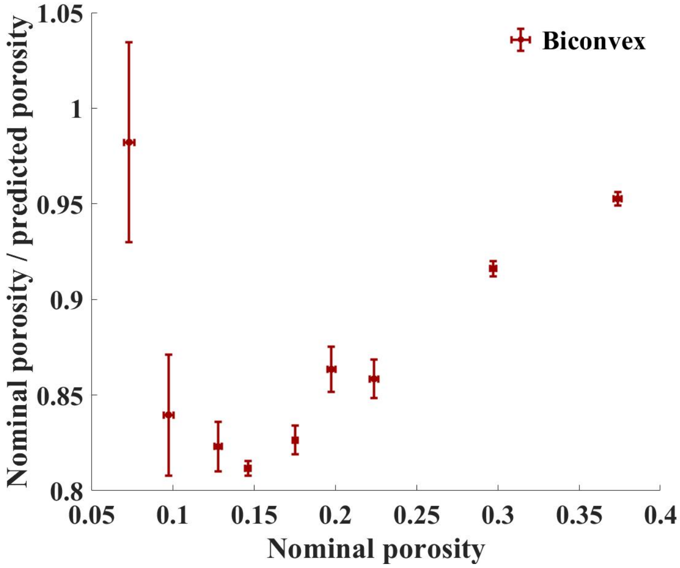

Figure 12: Estimated density distribution in biconvex tablets based on THz-TDS: The error bars indicate the standard deviation.
